# Collaborative framework on responsible AI in LLM-driven CDSS for precision oncology leveraging real-world patient data

**DOI:** 10.1038/s41698-025-01180-5

**Published:** 2025-12-04

**Authors:** Sonja Mathes, Dyke Ferber, Tobias Dreyer, Kai J. Borm, Luise Modersohn, Theresa Willem, Richard Dirven, Julien Vibert, Simon Kreutzfeldt, Raquel Perez-Lopez, Arsela Prelaj, Fredrik Strand, Richard D. Baird, Martin Boeker, Jakob Nikolas Kather, Maximilian Tschochohei, Jacqueline Lammert

**Affiliations:** 1 TUM School of Medicine and Health, Department of Dermatology, Technical University of Munich, Munich, Germany; 2 Institute for History, Theory and Ethics of Medicine, University of Mainz Medical Center, Mainz, Germany; 3 Else Kroener Fresenius Center for Digital Health, Technical University Dresden, Dresden, Germany; 4 National Center for Tumor Diseases, Heidelberg University Hospital, Heidelberg, Germany; 5 Department of Medical Oncology, Heidelberg University Hospital, Heidelberg, Germany; 6 TUM School of Medicine and Health, Department of Gynecology and Center for Hereditary Breast and Ovarian Cancer, Technical University of Munich, Munich, Germany; 7 German Cancer Consortium (DKTK), partner site Munich, a partnership between DKFZ and University Hospital of the Technical University of Munich (TUM), Munich, Germany; 8 Technical University of Munich, School of Medicine and Health, Department of Radiation Oncology, Munich, Germany; 9 Bavarian Center for Cancer Research (BZKF), Technical University of Munich Partner Site, Munich, Germany; 10 TUM School of Medicine and Health, Institute of Artificial Intelligence in Medicine and Healthcare, Technical University of Munich, Munich, Germany; 11 TUM School of Medicine and Health, Institute of History and Ethics in Medicine, Technical University of Munich, Munich, Germany; 12 Helmholtz Munich, Munich, Germany; 13 Department of Head and Neck Surgery and Oncology, Netherlands Cancer Institute, Amsterdam, The Netherlands; 14 Department of Otorhinolaryngology and Head and Neck Surgery, Radboud University Medical Center, Nijmegen, the Netherlands; 15 Cancer Core Europe, Villejuif, France; 16 Drug Development Department, Gustave Roussy, DITEP, Villejuif, France; 17 Division of Translational Medical Oncology, German Cancer Research Center (DKFZ), and NCT Heidelberg, Heidelberg, Germany; 18 Radiomics Group, Vall d’Hebron Institute of Oncology (VHIO), Barcelona, Spain; 19 Department of Medical Oncology, AI-ON-Lab, Fondazione IRCCS Istituto Nazionale dei Tumori (INT), Milan, Italy; 20 Department of Oncology and Pathology, Karolinska Institutet, Solna, Sweden; 21 Medical Diagnostics Karolinska, Karolinska University Hospital, Solna, Sweden; 22 Cancer Research UK Cambridge Centre, Cambridge, UK; 23 Department of Medicine I, University Hospital Dresden, Dresden, Germany; 24 Google Cloud, Munich, Germany; 25 TUM School of Medicine and Health, Center for Personalized Medicine (ZPM), University Hospital of the Technical University of Munich (TUM), Munich, Germany

**Keywords:** Cancer genetics, Cancer therapy

## Abstract

Precision oncology leverages real-world data, essential for identifying biomarkers and therapies. Large language models (LLMs) can aid at structuring unstructured data, overcoming current bottlenecks in precision oncology. We propose a framework for responsible LLM integration into precision oncology, co-developed by multidisciplinary experts and supported by Cancer Core Europe. Five thematic dimensions and ten principles for practice are outlined and illustrated through application to uterine carcinosarcoma in a thought experiment.

## Introduction

Large Language Models (LLMs) have transformed human perception of artificial intelligence (AI) primarily due to their ability to respond and reason in human language. The development of user-friendly interfaces has made LLMs accessible to non-technical users. In medicine, these advancements open opportunities, as LLMs can support clinicians by providing quick access to information, assist in clinical decision-making, and can take over simple, repetitive tasks such as documentation, thereby freeing up valuable time for healthcare professionals^1,2^. The capacities of LLMs extend beyond classical AI approaches that primarily analyze or classify structured data, as they combine content generation with the analysis and classification of unstructured data. More recently, multimodal models, which can process images and text simultaneously, have shown impressive results in simulated diagnostic scenarios^3,4^.

However, a recent systematic review found that only 5% of researchers utilize real-world patient data to train and evaluate LLMs, instead, most rely on synthetic datasets, which are easier to access and avoid data privacy concerns. Due to the limited real-world implementation of LLMs in clinical routines, little real-world evidence has been generated to confirm the theoretical potential of LLMs in real-world healthcare settings^5,6^. This current lack of evidence hinders the adoption of LLMs in routine clinical care. To fully unlock their potential for everyday healthcare, validation studies using real-world patient data are crucial to advance both the field and its clinical applications. Navigating the responsible use of real-world patient data for LLMs in complex health contexts benefits from practice-oriented guidelines to avoid uncertainties and navigate the responsible use of such data for LLMs in complex healthcare contexts.

In oncology, the challenges associated with using real-world patient data are particularly complex. Oncology clinical decision-making often requires integrating multimodal data into a comprehensive and individualized treatment plan. Currently, physicians in precision oncology carry out a range of labor-intensive tasks, such as creating and interpreting medical reports, annotating molecular mutations, manually curating scientific evidence, and interpreting biomarker profiles. These time-intensive processes, e.g., of translating genomic data into clinically actionable treatment plans^7^, delay the timely initiation of targeted therapies^8^, and thereby adversely impact patient outcomes.

**Fig. 1:**
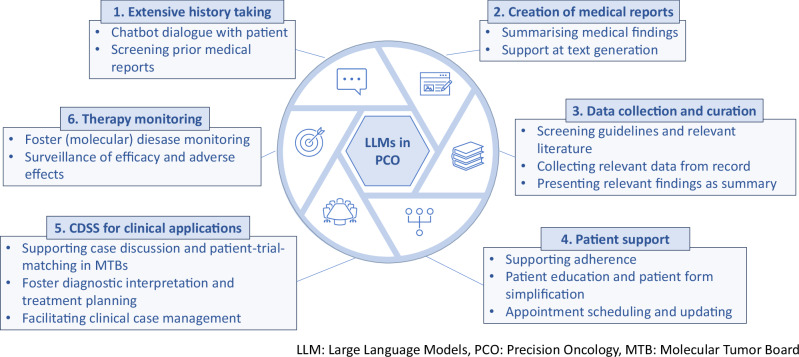
Applications of LLMs in Precision Oncology across the diagnostic and therapeutic process. LLMs can assist clinicians and related professions in the field of precision oncology with various tasks to eventually develop actionable treatment plans for patients. These models can be tailored to support time-consuming tasks, thereby improving resource management and enhancing the quality of care.

Clinical Decision Support Systems (CDSS) can leverage LLMs to overcome bottle necks in precision oncology: LLMs can extract structured information from unstructured patient data, enabling clinicians to make faster, more informed decisions (see Fig. 1). With this capability LLMs offer a range of applications across the diagnostic and therapeutic process in precision oncology, supporting tasks such as literature review, patient chart reviews, and biomarker interpretation, risk stratification, treatment recommendations and clinical trial matching (Fig. 1). These capabilities streamline workflows, enhance efficiency, and improve patient-centered care by minimizing delays in decision-making.

As LLMs become more prevalent in this field, it is essential to address both the advantages and limitations of using real-world data – an issue increasingly acknowledged by clinicians, patients, and policymakers.

Responsible AI (RAI) frameworks consider the ethical, legal, scientific, and clinical aspects necessary for the ethically sound development and deployment of AI^9,10^. They are essential to ensure the trustworthy application of AI in clinical settings due to the sensitivity of identifying data and the vulnerability of patients. As AI cannot be held accountable for clinical decisions, human operators retain accountability and may be legally liable for acting on AI-generated recommendations, especially when those recommendations do not align with established standard procedures^11^. This is crucial in precision oncology, where off-label treatments are often the only option for rare and treatment-resistant conditions lacking established guidelines.

To provide practice-oriented guidance for the responsible use of real-world data in LLMs for precision oncology, we designed a framework of five overarching dimensions to serve as a foundation for practical recommendations. Building on this, we developed ten principles to guide the practical implementation of LLM-based CDSS in precision oncology. We extend this with an example use case of a patient with uterine carcinosarcoma, a rare disease lacking established guidelines, where time-intensive manual research can be significantly accelerated using LLMs to improve patient outcomes. By providing practice-oriented guidance grounded in multidisciplinary experience and research, we aim to ensure that LLM-based CDSS leveraging real-world patient data not only achieve high standards of clinical efficacy but also adhere to ethical and legal requirements, ultimately bridging the gap between AI’s theoretical potential and its practical application in healthcare.

## Methods

Our literature review identified the need for specific recommendations on using real-world patient data responsibly in generative AI applications for CDSS in precision oncology. We then conducted an explorative literature review to identify a framework of dimensions for the responsible use of LLMs in CDSS in precision oncology. We subsequently held group discussions with an interdisciplinary team of ten experts in clinical oncology, AI, digital health, policy, and ethics, which led to the refinement of the five dimensions. Next, we identified ten practice-oriented principles based on our practical experience and insights from the literature, which should guide practitioners to implement all five dimensions.

**Fig. 2:**
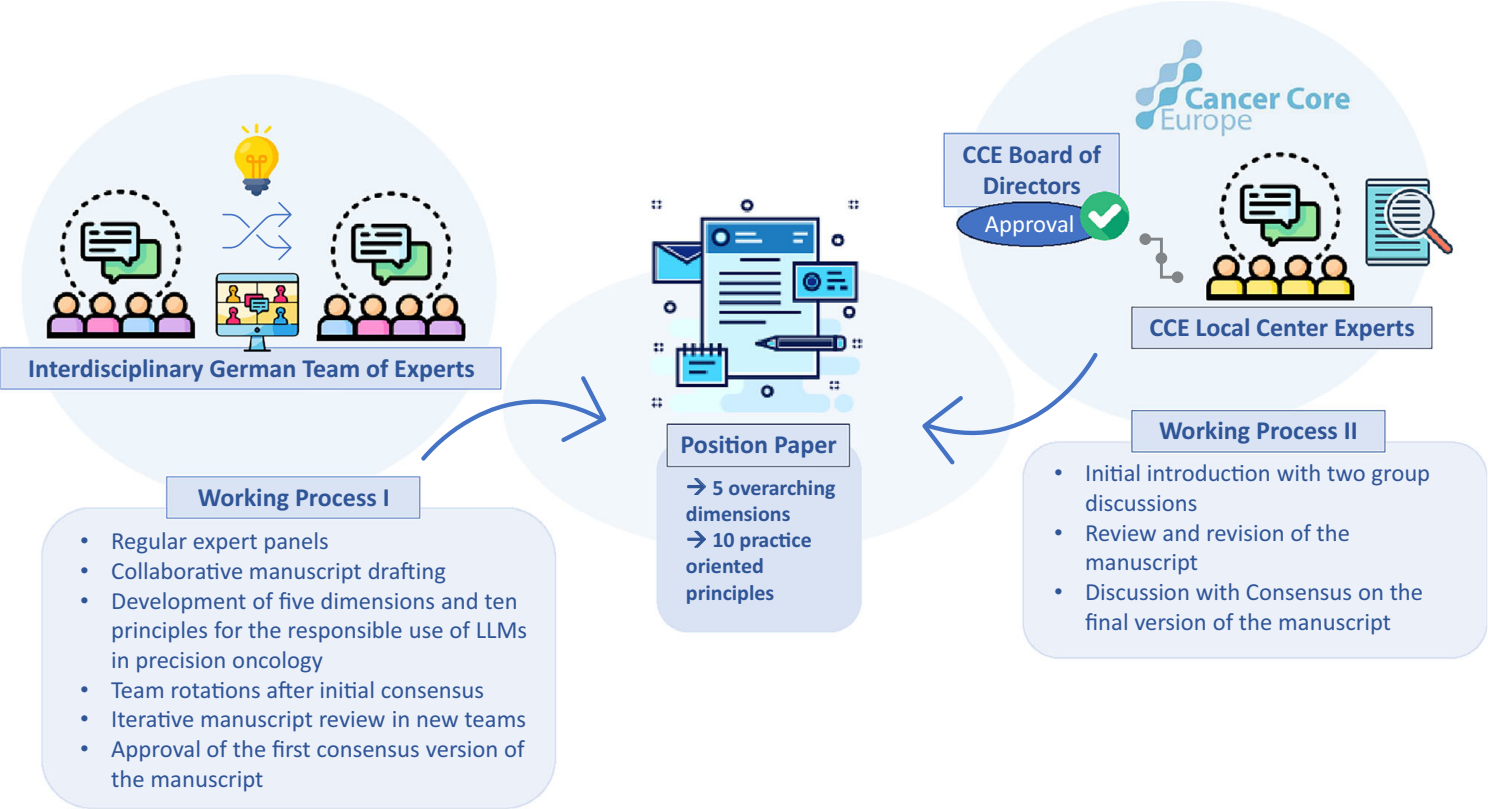
Methodology of manuscript compilation with the development of 5 overarching dimensions and 10 distinct practice-oriented principles. Structured development of a position paper on the responsible use of LLMs in precision oncology based on expert discussions and iterative consensus-building. An interdisciplinary German team of experts drafted five dimensions, each representing a thematic complex of responsible LLM-use for precision oncology with real-world data. From these more abstract complexes, ten practice-oriented, actionable principles were developed deductively through group discussions and repeated manuscript reviews. The manuscript underwent two working processes: initial drafting, team-based revisions, and consensus formation, followed by validation through discussions with local center experts within Cancer Core Europe (CCE). Final approval was granted by the CCE Board to ensure alignment with institutional and scientific standards.

**Fig. 3:**
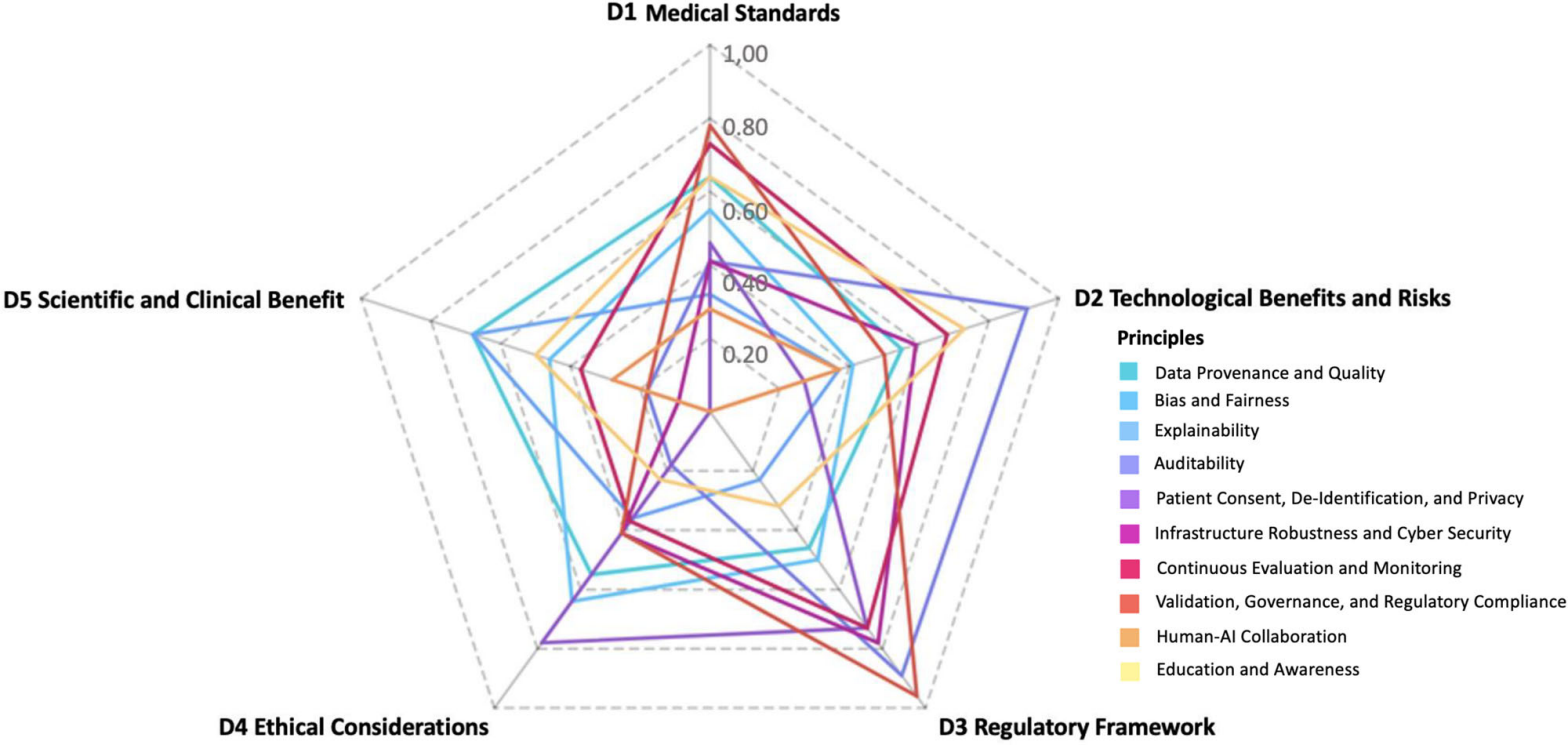
Mapping the influence of thematic dimensions on principle formulation based on a consensus survey of 11 experts. Evaluation of the ten user-oriented principles for applying LLMs in precision oncology based on their relevance across five thematic dimensions. Participating experts were asked to assess the contribution of each dimension to the respective principle: adherence to medical standards, technological benefits and risks, regulatory framework, ethical considerations, and scientific and clinical benefit, based on their professional experience. The mapping took place after the formulation of the principles was completed, to check for consensus on the relevance of the principle across thematic dimensions. For the mapping, the collection of aspects of each principle was rated dimension-wise for relevant overlap between principle and dimension by using a 5-point Likert scale.

To develop the principles, the group allocated each of the principles to a team of two authors according to their expertise. The principles were developed between March 2024 and August 2024. Regular consensus meetings allowed the group to discuss and agree on the content of the text sections. In July 2024, author groups were reassembled to review and edit the manuscript in new teams, providing a second perspective. We contacted Cancer Core Europe for external validation of the manuscript through renowned experts from their network working at the intersection of AI and precision oncology. Cancer Core Europe is a consortium of seven leading cancer centers across Europe, aiming to advance cancer research and improve therapeutic outcomes through collaborative, cross-institutional efforts. After meeting consensus within the team of ten, an additional seven authors from Cancer Core Europe (CCE)^12^ reviewed the manuscript between November 2024 and January 2025, revising each section until consensus was reached within the extended international team. To evaluate consensus on the relevance of the thematic dimensions for each principle, eleven available experts from our extended author group participated in an online survey (Google Forms) conducted after the formulation of the ten user-oriented principles. Using a 5-point Likert scale, they rated the extent to which each principle reflected aspects of the five thematic dimensions. The importance of each dimension within the respective principles was evaluated and measured quantitatively and is illustrated as a spider-plot in Fig. 3. After final review of the finalized text, figures, and tables by all contributing authors, CCE was asked to endorse the manuscript. We provide a detailed overview of the writing process in Fig. 2.

### Five dimensions of responsible AI for using real-world data with LLM for Precision Oncology

Our five dimensions represent large thematic complexes concerning the responsible use of LLMs with real-world patient data in precision oncology. For each dimension, we briefly summarize relevant concepts and considerations regarding the responsible implementation of generative AI with real-world patient data. We outline the relevance and scope of each dimension and highlight important contextual factors, including applicable regulations, standards, and guidelines.

### Dimension 1: Adherence to medical standards

Medical standards are striving to uphold a “standard of care”, as the established treatment that is beneficial for patients and subject to ongoing scientific evaluation^13^. Consensus on medical standards is, in part, summarized in medical guidelines. The constant evolution of medical standards is mirrored in the growing body of literature and the updating of medical guidelines. Generative AI in the form of LLMs holds the potential to contribute to the evolution of these standards by structuring previously inaccessible unstructured information, such as doctors’ letters, pathology reports, and clinical images for structured evaluation^14^ and facilitating the review of recent literature. There can be a bidirectional interaction between evolving medical standards and AI: On the one hand, principles of RAI can help ensure the implementation of medical standards in LLMs. On the other hand, LLMs can help advance medical standards, especially where they are undefined (e.g., in precision oncology). LLM systems must undergo critical evaluation of their adherence to medical standards, ensuring safety, efficacy, and relevance in real-world settings. This includes medical device certification, which ensures that LLMs in precision oncology comply with medical standards and safeguard patient safety.

### Dimension 2: Balancing technological benefits and risks

LLMs are able to quickly and efficiently extract information from unstructured text, image, audio, and video files, and create new, human-like content. This makes them valuable for managing the exponentially growing volume of unstructured and unlabeled information generated in Electronic Health Records (EHR) each day^15^. This is especially relevant for precision oncology, where analysis of extensive data sources becomes essential for tailoring treatments when standard guidelines fail or are nonexistent.

Technical designs need to carefully balance the benefits and risks of new technologies. For LLMs, this includes weighing their ability to analyze unstructured textual data quickly and reliably against shortcomings such as ‘hallucinations’, which are factually incorrect statements, that are hard to detect as such^16^. When technology leads to risks, these risks must be acknowledged and addressed^17^, also involving structured assessments, such as context-specific technology impact evaluations^18^.

### Dimension 3: Compliance with regulatory frameworks

Laws and regulations mandate medical standards and provide guidance on handling technology risk. Examples are the Medical Device Regulation (MDR) and the In Vitro Diagnostic Regulation (IVDR) in the European Union, and the Federal Drug Administration (FDA) and the Health Insurance Portability and Accountability Act (HIPAA) in the United States^19,20^. Additionally, the newly published EU AI Act mandates implementation of controls for AI-products, based on the risk level of their application contexts^21^. Many LLM-based use cases in medicine fall under the “high risk” category as they support clinicians and patients to make healthcare decisions. Any modification or in-house operation of a general-purpose LLM can lead to clinics becoming AI system providers^22^. While the AI Act gradually comes into full effect by 2027, business and IT decision makers in healthcare institutions were required to become familiar with the obligations of AI system providers and implement relevant governance frameworks and controls already by August 2026^23^.

### Dimension 4: Respecting ethical considerations

Ethical considerations form another thematic complex, inherently relevant to the responsible use of LLMs in precision oncology. Ethical standards and guidelines in medicine give the highest priority to promoting patient well-being^24–28^. Historical examples underscore the importance of critical ethical guidance in research and development at this^29^. In AI for precision oncology, AI-specific novel ethical frameworks are increasingly established^30–33^, while a broad range of ethical principles has already been articulated in the existing literature^31,34^. New ethical considerations and principles add perspectives, while traditional perspectives remain valuable, e.g., reflecting the four principles of biomedical ethics by Beauchamp and Childress^27,32^. Novel near-term and long-term ethical concerns have been named, and including bias, fairness as important considerations, calling for responsible AI^9^. As a new concept developing ethical standards for the creation of responsible AI-systems, RAI has emerged from related scientific fields^35^. To ensure that algorithms comply with ethical requirements, embedding ethicists in development teams has been suggested^36^. In the context of precision oncology, the rapid technological evolution of LLMs continues to raise new ethical questions, especially as the race for advances in AI accelerates. Our team of authors proposes incorporating established ethical views alongside emerging ethical considerations when developing LLMs that utilize real-world patient data.

### Dimension 5: Providing scientific and clinical benefit

The increase of precision in medicine is data-driven, continuously integrating insights from biomedical research and health information. By structuring, synthesizing, validating, and analyzing this abundance of information across fragmented data sources, AI can substantially contribute and thereby advance precision medicine across healthcare sectors^37–40^ and accelerate scientific innovation^4,41^. Aims to prevent risks of AI in research and clinical applications, like dual-use potentials and data-safety concerns, are mirrored in extensive governance frameworks, such as the GDPR^42^, EU AI Act^21^, or DSA/DMA, partially hindering technological advancement^43^. Additional considerations for clinical usability include social and financial factors, such as fostering public acceptance through explainability and education, and calculating the increasing costs of in-depth testing and refinement for adequate monitoring^43^. These factors are particularly relevant in the healthcare sector, where a substantial portion of funding comes from social systems. While the successful implementation of LLMs in science and clinical routine might eventually yield scientific and clinical benefits, including returns on investment, increased accessibility of precision oncology for patients^44,45^ and potentially incentivizing further LLM-centered research, these factors must be carefully weighed.

### Ten principles as practical guidance for the responsible use of LLMs in precision oncology

Drawing on the content identified as relevant within the thematic dimensions, we proceed to curate ten user-oriented principles for practical guidance. Each principle presents a concise collection of relevant aspects from the dimensions to aid at the actual application of generative AI with real-world patient data in precision oncology. The contribution of the overarching dimensions to each respective principle, as assessed by the experts in our author team, is mapped in Fig. 3. To provide relevant practice-oriented guidance, we contextualize collections of relevant aspects within current challenges related to the practical implementation of LLMs in precision oncology use cases involving real-world patient data. We intentionally permit individual aspects from the thematic dimensions to be addressed across different principles, as the thematic dimensions may offer different facets, depending on the principle’s narrower thematic focus, all of which can contribute to directive recommendations.

### Principle 1: Data provenance and quality

Data provenance is defined as “information about entities, activities, and people involved in producing a piece of data or thing, which can be used to form assessments about its quality, reliability or trustworthiness”^46^. Accordingly, LLMs, like other forms of ML systems, leveraging real-world patient data for precision oncology, require rigorous standards for reporting on data sources, data composition, training data, data collection, data quality metrics as well as bias and variability, including data sources and demographic information on patients (see Table 1 for detailed recommendations).

**Table 1:** Suggestions to support adequate Data Provenance and Quality for applying generative AI to real-world patient data

***Data Sources***	• Specify origins and types of datasets used with inclusion of primary care data (e.g., electronic health records, clinical trials, and registries) as well as generic medical data (textbooks, PubMed, clinical coding systems).
***Dataset Composition***	• List the total number of individuals, cases, or records, and duplicates included in the dataset. • Clearly describe inclusion and exclusion criteria and limitations of the dataset. Consider distinct characteristics and differences between vendor-level LLM training requirements and hospital-specific application in emphasizing differing challenges in data provenance.
***Data Quality Metrics***	• Disclose detailed dataset statistics like distribution of data types (such as clinical notes, imaging, laboratory values) and descriptive statistics of data missingness. Provide details of the deidentification pipeline use. • Up-to-date, indicating the collection period from start to end or details on coding systems and ontologies employed, such as ICD-10 or SNOMED-CT.
***Demographic Information***	• Inform on geographic locations from which the data was collected. • Represent regional variations in disease prevalence, treatment approaches, and healthcare access in the data (health equity). • Ensure representation of ethnic minority and marginalized populations.
***Bias and Data Variability***	• Provide descriptions of all measures taken to detect and correct for biases in the data, addressing issues like over- or underrepresentation of specific groups.
***Legal Requirements***	• Main privacy requirements in compliance with HIPAA (US) or GDPR (EU), including consent for the incorporation of non-anonymized data into datasets. • Disclose details on data licensing and reuse terms for any published literature data used in model training.
***Ethical aspects***	• Detail any ethical approvals obtained for the use of data, especially for the use of sensitive data.

This table outlines key recommendations for establishing robust data provenance and quality standards in the use of generative AI, like LLMs, for precision oncology. It addresses critical elements including the specification of data sources (e.g., electronic health records, clinical trials, registries, and general medical data), detailed documentation of dataset composition (such as case counts, inclusion/exclusion criteria, and application-specific nuances), and reporting of data quality metrics (including data type distributions, temporal coverage, and coding systems used). Additionally, it highlights the importance of systematically identifying and mitigating biases and data variability to support fair and reliable AI applications. These components form the minimal requirements for ensuring data provenance and quality for responsible use of real-world patient data in AI-driven clinical decision support.

Collecting provenance and quality information of all data used as input for inference, e.g., from information systems or prompts, their pre-processing steps, like de-identification, normalization, or coding/annotation, and corresponding results, are minimal necessary requirements for inference audit trails.

### Principle 2: Bias and fairness

Fairness in connection with avoidance of bias is are core value of medical ethics^24,27^ and therefore a critical concept to consider for responsible use of LLMs in precision oncology. Both aim to achieve increased transparency and equitable healthcare outcomes across diverse populations. At this, fairness also emphasizes health equity, implying fair access to health resources from the outset. In computational health, diverse definitions of fairness are used^47^, with many criteria for fairness being observational, such as examining the distribution of factors to decide about access to precision oncology^47^. Concepts of fairness are often conflicting, e.g., conflicts between individual and group fairness^47^ necessitating balancing principles of group fairness, like demographic parity (distribution of resources based on a singular criterion, e.g., gender), with fairness from the individual’s point of view. However, this approach of deliberate differentiation between groups can result in disadvantages for singular individuals, which can be addressed by mechanisms like equality of odds^48^. Apart from known group differentiators, subconscious discrimination in training data for models can lead to algorithmic bias, necessitating critical interpretation, evaluation, and monitoring, including limiting use to suitable application contexts and implementing regular, sample-based human reviews of AI suggestions^49^.

### Principle 3: Explainability

Maximizing the explainability of generative AI enhances transparency and fosters trust among medical professionals. Many AI models, particularly deep learning architectures, are often criticized for their “black box” nature^22^, which is critical in the sensitive context of precision oncology, when the rationale behind their predictions cannot be tracked by the responsible clinicians.

To address this challenge at least partially, researchers have developed explainable AI (XAI) techniques helping users understand why an AI model came to a certain conclusion^50,51^.

A notable example is a study by Kuenzi et al., which demonstrated the potential of XAI in precision oncology by developing an interpretable deep learning model for predicting drug responses in cancer patients. This model achieved high accuracy and provided explanations traceable and consistent with existing biological knowledge, thereby enhancing its clinical utility^52^.

However, trade-offs between explainability and model performance should be acknowledged^53,54^. Inherently explainable models can be limited by their susceptibility to confounders, while post-hoc explainability techniques often fail to transparently reveal the reasoning behind a model’s decisions, potentially misleading users. These methods lack performance guarantees, frequently rely on approximations, and may even reduce model accuracy. Crucially, while XAI methods provide *descriptive* insights into model behavior, they do not offer *normative* evaluations of whether that behavior is justified or appropriate. Attempting to bridge this gap through intuition risks introducing additional biases. Moreover, explainability can paradoxically foster over-reliance on AI systems, potentially reducing user vigilance and critical evaluation. To ensure the safety, efficacy, and fairness of AI systems, thorough validation across diverse populations remains the most reliable approach. XAI should primarily serve as a tool for developers and auditors to probe and refine models, rather than as a justification for individual decisions^55^. Therefore, caution is warranted when interpreting explainability outputs as indicators of clinical or operational correctness. For generative AI models, XAI can involve citing the sources of their recommendations and enabling users to validate outcomes. Techniques like Retrieval Augmented Generation (RAG) technically provide relevant documents as lookup data for the model and plays a crucial role in advancing the explainability, reliability, and auditability of AI-assisted clinical decision-making^1^.

### Principle 4: Auditability

Auditability is crucial for ensuring the accountability of AI systems in precision oncology. It is also a key requirement in much of the prevailing regulation governing the use of AI in medicine (see Regulatory Frameworks). This involves the ability to track and review the decision-making process of AI models, identify potential sources of error, and correct them promptly. To achieve auditability, AI systems should incorporate logging and monitoring mechanisms that systematically capture relevant data throughout the decision-making process, such as source document references, model outputs, and precise timestamps. This data can then be analyzed to assess the performance of the model, detect anomalies, and pinpoint areas for improvement^56^.

### Principle 5: Patient consent, de-identification, and privacy

Since the latter half of the 20th century, patient consent and privacy considerations have been considered essential for respecting patient autonomy and physician beneficence^57^.

The clinical and scientific use of genetic data is especially sensitive due to its highly personal nature. Meaningful consent to use genetic data requires patients to understand associated privacy concerns and must enable them to make informed decisions^58^. To mitigate patient privacy concerns reasonably, anonymization and pseudonymization are common data protection methods from the General Data Protection Regulation (GDPR)^59^. Pseudonymization, relying on cryptographic algorithms or lookup tables, renders patients theoretically still identifiable^59^. Anonymization, involving removing all personally identifiable information (PII), is preferred with respect to data protection for patients, as it theoretically renders re-identification impossible^60^. However, true anonymization is challenging, especially for mutational information, since indirect identifiers like age, location, and family and medical history can still enable reidentification^61^. Simply omitting these data points may lead to increased information loss^62,63^. Thorough selection of anonymization strategies may contribute to acceptable information loss, only mildly affecting data utility^62,63^. Anonymization methods should be evaluated across diverse data streams and demographic groups, with detailed documentation of the applied techniques.

Local operation of AI models and protected data storage may enhance data protection, even if full anonymization is not feasible^54^. Technological evolution may necessitate clarifications and adaptations of data protection regimes where appropriate, especially if they only add complexity without enhancing data security.

### Principle 6: Infrastructure Robustness and Cyber Security

For the protection of patient data and patient well-being robust infrastructure is a key principle of applying generative AI to real-world patient data, necessitating cybersecurity safeguards and regular internal and external audits. These requirements are enforced by the current regulation^21^. In accordance with data minimization, only data that is strictly necessary for the given use case should be stored^59^. Practitioners need to ensure that they use tested and validated software and infrastructure and implement robust governance, risk, and compliance (GRC) frameworks. Operators of an AI system need control over stored data, and local data processing (without internet access) is therefore preferable. For good research practice, adherence to FAIR principles (Findable, Accessible, Interoperable, Reusable) is beneficial^64,65^.

### Principle 7: Continuous evaluation and monitoring

In the SARS-CoV2 pandemic, quickly emerging epidemiological changes in health data were seen, leading to dataset shifts. AI may struggle to recognize such deviations, requiring human supervision to ensure AIs work in situations that differ from their training situations^66^. Due to the highly sensitive applications of generative AI in precision oncology, continuous, multifaceted evaluation and monitoring are required, considering the specifics of the respective application context^67^.

Molecular Tumor Boards (MTB) are a key element of precision oncology^68^ and see benefits from integration of AI in the field, e.g., for literature search and synthesis^69^. Legal requirements such as the GDPR^42^ and EU AI Act^21^, regulate generative AI applications in precision oncology, mandating mechanisms for evaluation and monitoring for high-risk applications. These expectations, next to evaluating real-world performance, can be met by implementing technical measures for monitoring the performance of (generative) AI applications, leveraging established metrics such as Ragas^70^ or BERTScore^71^. Additionally, AI system recommendations should be reviewed by clinicians (human-in-the-loop), with comprehensive sample-based reviews^34,72^.

### Principle 8: Validation, governance, regulatory compliance

AI has increasingly been focused on as a topic in jurisdiction, with growing regulations acknowledging this trend^22^. The EU AI Act is a landmark in international regulation, becoming mandatory in August 2026^21,73^. Complexity of legal requirements, particularly in data protection^74^, complicate processes for researchers and may incentivize the development of lower-risk medical AI-devices rather than complex solutions^22^.

Thorough validation is essential before integrating LLMs into clinical workflows, as incorrect AI outputs may lead to considerable risks for individuals^75^. This has led to calls for transparency and accountability in AI prosecution^76^. Current literature on liability discusses factors influencing physicians’ accountability, such as increased responsibility when following more unconventional AI recommendations^11^. Edge cases like this may arise more naturally in precision oncology, where standard treatments are either exhausted or unavailable^77^. Researchers suggest resolving these issues by allowing for moral customization of AI settings by patients, returning autonomy to the individual^78^. Quick responses of legislators to trends and innovations may be crucial to protect patients, their families, clinicians, and researchers from uncertain legal situations.

### Principle 9: Human AI collaboration

Humans and AI are projected to increasingly collaborate on clinical tasks^79,80^. As AI solutions become more advanced, it is crucial to ensure that these technologies respect human uniqueness and dignity, including awareness on the influences of AI on users^31^. This implies effective and secure integration of AI applications in clinical routine, enhancing, rather than replacing, the expertise of physicians and providing explainable and responsible AI applications for patients^30–33^. Currently established principles lack clarity in guiding Human AI Collaboration^30^. To achieve an appropriate balance between uncritical acceptance of AI proposals and overt distrust of beneficial AI applications^81^, AI outputs require human validation during model development and in every specific case of use.

Currently, AI validation and training rely heavily on human-annotated data and feedback, especially for LLMs as they generate non-reproducible, non-deterministic output^82,83^.

Risks can be mitigated with “human-in-the-loop” refinement^69^. In precision oncology, this implies ensuring alignment with clinical requirements and by integrating physician specialist domain knowledge. This requires fine-tuning, additional prompt engineering, or incorporating trusted (online-) data sources via RAG^84^ or In-Context Learning^85^.

### Principle 10: Education and Awareness

Even though easy and comfortable to use^86,87^, AI applications in vulnerable patient groups, like in precision oncology, necessitate specific education for all stakeholders, rather than leaving their responsible use primarily to interested early adopters in the field, creating awareness for potential risks as well as opportunities. Education can leverage AI for teaching directly, enabling hands-on learning concepts, together with immersive learning methods, which have been shown to outperform established teaching concepts^88^. Education of patients, physicians, and researchers on AI is crucial for valuable and responsible integration of LLM in clinical applications, and physicians’ training should meet the standards of medical education^89^. This comprises learning on and through AI-tools in three primary ways: directly in the teaching process, as a supplementary tool for educators, and by empowering physicians in training^90^. Since interactions with AI may involve real-world data and personal information of all participants, effective measures for data and privacy protection are essential to ensure learners’ legal integrity^91^.

### Thought experiment: Blueprint for LLMs in Clinical decision support systems for precision oncology

To validate the usability of the ten principles for the application of generative AI for precision oncology comprehensively, we have applied them to a Rare Gynecological Tumor (RGT) use case.

RGTs present a major challenge in oncology. In gynecology, they account for over 50% of all malignancies and include more than 30 distinct histologic subtypes for this specialty alone, with an estimated 80,000 new cases annually in Europe^92^. The rarity of RGTs, with an incidence of less than 6 per 100,000 women, due to limited patient numbers for each subtype, hinders the development of robust clinical practice guidelines. Consequently, treatment decisions often rely on less standardized approaches, such as retrospective studies, case reports, expert opinions, or extrapolating data from analogous tumors in other anatomical sites^93^.

Uterine carcinosarcoma (UCS) exemplifies the challenges in managing rare tumor entities. Despite established endometrial cancer treatment protocols, UCS remains a distinct, aggressive malignancy with limited therapeutic options. This RGT is frequently excluded from clinical trials, contributing to a poor prognosis characterized by low survival rates and high recurrence. Advanced UCS management is further complicated by treatment resistance, lack of standardized second-line therapies, and poor outcomes with subsequent treatment lines^94^. While the molecular characterization of UCS offers potential therapeutic targets, translating genomic data into effective clinical strategies remains challenging. Here, we want to introduce LLM-enabled CDSS applications with the potential to improve disease management in a UCS patient who has progressed on first-line therapy.

We provide a case-based workflow, especially underscoring the critical role of real-world patient data integration in the development and refinement of LLMs for precision oncology.

### Thought experiment and Workflow for the responsible use of LLMs in Precision Oncology

Upon admission to an oncological care unit of a UCS-patient with progressive disease after first-line treatment, patient data would be collected from existing records and through a detailed anamnesis, ensuring adequate data provenance. Meaningful Informed Consent should be obtained from the UCS patient, ensuring understanding of the relevant risks and benefits of Molecular Profiling and LLM use.

Annotation of real-life data and thorough literature research, together with (AI-supported) diagnostic assessment, is necessary at this step. This process is time-consuming, resource-intensive, and prone to suboptimal patient care due to the absence of standardized treatment guidelines and the challenges associated with manual literature review. At this, a trained professional can leverage an LLM-enabled CDSS - respecting medical standards, data protection requirements, as well as ethical requirements to alleviate these tasks. By instantly analyzing vast amounts of real-world patient data, streamlining literature reviews, and aiding in the generation of treatment plans, a CDSS offers a potential solution to overcome the existing bottlenecks at this point and improve patient care.

**Fig. 4:**
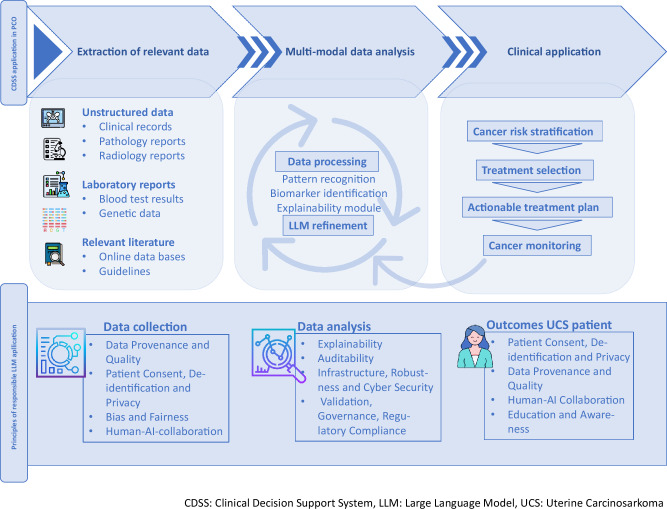
Flowchart for Integration of Responsible Use of LLMs in Decision Support Systems (CDSS) with Real-World Patient data for PCO-Patients, exemplified for Patients with Uterine Carcinosarcoma. Structured framework (blueprint) to the responsible integration of LLMs in precision oncology workflows. LLMs are applied across data extraction, multi-modal data analysis, and clinical decision-making to process laboratory reports, unstructured clinical data, and literature, support data analysis and explainability, and assist in cancer risk stratification, quality, patient privacy, explainability, auditability, and regulatory compliance, reflects their contextual relevance across workflow components to ensure responsible LLM deployment in clinical and research settings.

The developed treatment suggestions from the annotation process facilitated through this approach form the basis for Shared Decision-Making (SDM) on identified treatment options. At this step, patients must be informed and educated about the use of CDSS and the therapeutic options. LLMs can support patient–trial matching, if trials are available, or, otherwise, can assist in reviewing current evidence to suggest treatments, including potential off-label options for rare diseases. The paucity of UCS-specific clinical trials usually necessitates off-label treatment decisions based on the available evidence. Therefore, inclusion into clinical trials/a Cost Coverage Request is issued, again with the help of an LLM-enabled CDSS, and finally, an actionable treatment plan is developed. The treatment plan is executed, and the documented results are analyzed with the support of LLMs that strictly adhere to legal and ethical requirements, avoid bias to the extent possible, and enable clinicians and researchers to interact in a responsible, efficient way with the AI. Clinical outcomes are documented using LLMs that strictly follow data protection and ethical requirements. The resulting multimodal data of a variety of patients is then analyzed and used to enhance LLM performance in supporting effective UCS treatment. This blueprint use case of a gynecological precision oncology patient with uterine carcinosarcoma serves to highlight a framework for the application of similar methodologies to refractory diseases for other fields of precision oncology. The exemplary workflow is illustrated in the flowchart in Fig. 4.

With this thought experiment, we demonstrate how rigorously applying our design principles for RAI in CDSS ensures that an LLM will address all five dimensions of RAI in precision oncology with real-world data. The ensuing system adheres to medical standards, balances technology benefits and risks, complies with regulatory frameworks, respects ethical considerations, and ensures that the system will provide benefits to patients, clinicians, and the scientific community. A detailed checklist for the application of RAI in comparable Precision Oncology settings is provided in the Supplement (Supplement 1).

In our thought experiment, we propose a workflow that operationalizes our framework of five dimensions and ten practice-oriented principles for real-world applications. The UCS patient case can be readily replaced by anonymized real-world cases, such as those from The Cancer Genome Atlas (TCGA) UCS cohort established in the United States^95,96^.

While we highlighted the principal applicability of our framework in precision oncology contexts, we note the limitations of our framework. The thematic dimensions may be broadly transferable to other domains of precision medicine, such as neurology; the principles reflect context-specific guidance tailored to precision oncology. Further refinement and adaptation will be required to apply the principles to other medical fields. Also, given the continuous evolution of the field, critical engagement with ongoing developments within the context of this framework remains essential.

### Conclusion

Generative AI in oncology holds potential for improving patient, scientific, and medical outcomes. Responsibly integrating real-world patient data is required to fully realize this potential. Recent advancements have made AI more accessible and user-friendly, but its responsible use in precision oncology requires comprehensive education for all stakeholders. This review explores five dimensions for AI implementation: adherence to medical standards, promotion of scientific and medical benefits, consideration of technological advantages and risks, regulatory frameworks, ethical considerations; as well as ten guiding principles for the application of generative AI in precision oncology: Data Provenance and Quality, Bias and Fairness, Explainability, Auditability, Patient Consent, De-identification and Privacy, Infrastructure Robustness and Cybersecurity, Continuous Evaluation and Monitoring, Validation, Governance and Regulatory Compliance, Human-AI Collaboration, Education and Awareness. These principles will assist practitioners in integrating generative AI within LLMs for precision oncology, using real-world patient data in alignment with RAI principles.

## Supplementary information

Supplement 1 Mathes et al.

## Data Availability

No datasets were generated or analyzed during the current study.
