# Supplementary material for: Collaborative framework on responsible AI in LLM-driven CDSS for precision oncology leveraging real-world patient data
Source: NPJ Precis Oncol. 2025 Dec 4;10:15. doi: 10.1038/s41698-025-01180-5 (PMC12796327; doi:10.1038/s41698-025-01180-5)
Supplement: Supplementary file 1 — Supplement 1 Mathes et al. [file 41698_2025_1180_MOESM1_ESM.docx]

*Supplement 1: Checklist applying RAI principles to LLM in CDSS in Precision Oncology*

| **RAI** | **Application for LLM in CDSS for Precision Oncology treatment suggestions** |
| --- | --- |
| Data Provenance and Quality | ● Define data quality criteria  ● Ensure data meets quality criteria before ingestion  ● Keep clear record of metadata, e.g., source system, created date, data classification |
| Bias and Fairness | ● As part of data quality criteria, include qualitative measures of bias and fairness, e.g., demographic distribution  ● Ensure that bias in data is corrected through appropriate controls such as manual adjustments or automated algorithms |
| Explainability | ● Provide clear and transparent explanation of configuration of the AI system  ● Provide attribution of source data that led to AI system recommendations where appropriate, e.g., without exposing PII |
| Auditability | ● Establish audit trails for AI-generated recommendations  ● Conduct regular audits to ensure the AI system complies with clinical and regulatory standards. |
| Patient Consent, De-Identification, and Privacy | ● Educate patients on data usage and data privacy, then obtain explicit patient consent for data usage in AI system  ● De-identification is not feasible with small number of available features, so need to ensure appropriate protection of PII  ● Implement strong, privacy-first technical design and appropriate controls such as strict access management, encryption, logging and audits |
| Infrastructure Robustness and Cybersecurity | ● Implement strong cybersecurity controls aligned to data classification, e.g., NIST IR 8432  ● Implement AI system in client-server architecture in self-managed data centers, ensure strong encryption of data-at-rest and in-transit, ensure compliance with business continuity policies |
| Continuous Evaluation and Monitoring | ● Implement a mixture of automated and manual controls to monitor and evaluate the AI systems performance against user requirements, e.g., accuracy and loss monitoring, and sample checks of answers |
| Validation, Governance, Regulatory Compliance | ● Ensure that the AI system and provider model is tested against industry standards, e.g., ISO/IEC 25059  ● Validate your implementation and the expected outcomes in a suitable study according to scientific principles  ● Establish appropriate governance bodies to monitor the performance of the AI system as per prevailing regulation, e.g., EU AI Act |
| Human-AI Collaboration | ● Train healthcare professionals on how to effectively use AI tools in UCS treatment planning  ● Effectively integrate AI in clinical routine workflows in UCS diagnosis, treatment planning and monitoring  ● Ensure that every treatment recommendation is reviewed by a human expert (human-in-the-loop) |
| Education and Awareness | ● Train clinicians, patients, and stakeholders about the capabilities and limitations of AI in UCS treatment  ● Promote awareness of responsible AI practices to build trust and acceptance among users |
